# 2-Ketoglutarate-Generated In Vitro Enzymatic Biosystem Facilitates Fe(II)/2-Ketoglutarate-Dependent Dioxygenase-Mediated C–H Bond Oxidation for (2*s*,3*r*,4*s*)-4-Hydroxyisoleucine Synthesis

**DOI:** 10.3390/ijms21155347

**Published:** 2020-07-28

**Authors:** Xiao-Ran Jing, Huan Liu, Yao Nie, Yan Xu

**Affiliations:** 1 Key Laboratory of Industrial Biotechnology of Ministry of Education and School of Biotechnology, Jiangnan University, 1800 Lihu Road, Wuxi 214122, China; rushjxr794@163.com (X.-R.J.); 18635924851@163.com (H.L.); 2 State Key Laboratory of Food Science and Technology, Jiangnan University, 1800 Lihu Road, Wuxi 214122, China

**Keywords:** Fe(II)/2-ketoglutarate-dependent dioxygenase, 2-ketoglutarate generation, regio- and stereo-selective synthesis, hydroxy amino acids, sequential cascade reaction

## Abstract

Fe(II)/2-ketoglutarate-dependent dioxygenase (Fe(II)/2-KG DO)-mediated hydroxylation is a critical type of C–H bond functionalization for synthesizing hydroxy amino acids used as pharmaceutical raw materials and precursors. However, DO activity requires 2-ketoglutarate (2-KG), lack of which reduces the efficiency of Fe(II)/2-KG DO-mediated hydroxylation. Here, we conducted multi-enzymatic syntheses of hydroxy amino acids. Using (2*s*,3*r*,4*s*)-4-hydroxyisoleucine (4-HIL) as a model product, we coupled regio- and stereo-selective hydroxylation of l-Ile by the dioxygenase IDO with 2-KG generation from readily available l-Glu by l-glutamate oxidase (LGOX) and catalase (CAT). In the one-pot system, H_2_O_2_ significantly inhibited IDO activity and elevated Fe^2+^ concentrations of severely repressed LGOX. A sequential cascade reaction was preferable to a single-step process as CAT in the former system hydrolyzed H_2_O_2_. We obtained 465 mM 4-HIL at 93% yield in the two-step system. Moreover, this process facilitated C–H hydroxylation of several hydrophobic aliphatic amino acids to produce hydroxy amino acids, and C–H sulfoxidation of sulfur-containing l-amino acids to yield l-amino acid sulfoxides. Thus, we constructed an efficient cascade reaction to produce 4-HIL by providing prerequisite 2-KG from cheap and plentiful l-Glu and developed a strategy for creating enzymatic systems catalyzing 2-KG-dependent reactions in sustainable bioprocesses that synthesize other functional compounds.

## 1. Introduction

The C–H functionalization of small molecules is an important reaction in organic synthesis [1,2]. The oxidized products were used as building blocks in pharmaceutical syntheses [3]. The C−H bond activation by biocatalysis has been reported to date, with less environmental impact compared with conventional counterparts [4]. Fe (II)- and 2-ketoglutarate-dependent dioxygenases (Fe(II)/2-KG DOs) catalyze various C–H-activation-mediated reactions including hydroxylation, deoxygenation, desaturation, and ring extension [5]. Recently, research attention has been directed to the asymmetric hydroxylation of inactivated carbon atoms via direct C–H bond functionalization [1]. Amino acids are substrates of Fe(II)/2-KG DOs and are usually converted into hydroxy amino acids [6]. Various amino acid hydroxylases producing hydroxy amino acids have been characterized among the Fe(II)/2-KG DOs. These include lysine hydroxylase [7], asparagine hydroxylase [8], l-Ile dioxygenase (IDO) [9], leucine hydroxylase [10], and proline hydroxylase [11,12].

Hydroxy amino acids have numerous applications in the pharmaceutical and food industries. They serve as chiral building blocks and food additives [13]. For example, *trans*-4-hydroxy-l-proline (*trans*-4-Hyp) is a major component of collagen [14]. It is a crucial precursor in the pharmaceutical synthesis of *n*-aryl pyrrole [15] and (−)-kainic acid [16]. Moreover, (2*s*,3*r*,4*s*)-4-hydroxyisoleucine (4-HIL) naturally occurs in *Trigonella foenum-graecum* (fenugreek) seeds, accelerates insulin secretion, and could be administered to treat type II diabetes [17]. In addition, 4-hydroxynorvaline isolated from *Lathyrus odoratus* seeds [18] stimulates insulin secretion and has antidiabetic properties [19]. Further, 5-hydroxyleucine participates in griselimycin synthesis. Griselimycin may have efficacy against drug-resistant *Mycobacterium tuberculosis* and strong antituberculosis activity [10].

Fe(II)/2-KG-DOs catalyze C–H oxidation using 2-KG as a co-substrate. Thus, the amount of 2-KG directly influences the catalytic efficiency of Fe(II)/2-KG DOs. Several studies applied metabolic engineering to generate 2-KG and facilitate Fe(II)/2-KG DO-mediated C–H bond oxidation. Smirnov et al. redirected the tricarboxylic acid (TCA) cycle intermediate 2-KG to biotransform l-Ile into 4-HIL using IDO. They achieved 82% yield using 100 mM l-Ile as the substrate [20]. Zhang et al. optimized the TCA cycle by dynamically modulating the activity of the 2-ketoglutarate dehydrogenase complex in *Corynebacterium glutamicum.* In this manner, they generated ample 2-KG and yielded 232.52 mM of 4-HIL after 64 h [21]. A similar strategy was applied to other Fe(II)/2-KG-DOs-mediated reactions in order to overcome insufficient 2-KG supply. Lin et al. reported a deacetoxycephalosporin-C synthase (DAOCS) that converted penicillin substrates into cephalosporins. They reconstituted the TCA cycle to force 2-KG into an enzymatic reaction [22]. However, this modification had a negative effect on cell growth [21,22], low conversion efficiency, and long reaction time. In contrast, 2-KG has also been produced by chemical synthesis and enzymatic oxidation [23,24]. Enzymatic 2-KG syntheses were conducted under relatively mild reaction conditions, consumed minimal energy, and produced little pollution. Liu et al. reported the use of l-amino acid oxidase (LAAO) for 2-KG production with l-glutamic acid (l-Glu) as the substrate. Nevertheless, LAAO had low activity towards l-Glu and was inhibited by high substrate and product concentrations. l-glutamate oxidase (LGOX) has been identified with high l-Glu-to-2-KG conversion activity without requiring exogenous (flavin adenine dinucleotide) FAD [25]. Since the oxidative deamination from l-Glu to 2-KG is along with H_2_O_2_ generation, Wu et al. developed a cascade strategy of co-expression LGOX and catalase (CAT) to eliminate H_2_O_2_ [26].

In vitro and in vivo (whole-cell) enzymatic syntheses of complex molecules have been successfully implemented in recent years [27]. Here, we implemented an in vitro three-enzyme system to synthesize hydroxy amino acids. We used IDO to hydroxylate l-amino acids and performed oxidative decarboxylation of 2-KG to succinate (SA) [28]. As the consumption of superstoichiometric amount of 2-KG is one of the limitations of the system, an adequate supply of 2-KG derived from inexpensive substrates is necessary for an in vitro system. Hence, 2-KG generation from l-Glu catalyzed by LGOX was adopted in combination with CAT to eliminate the H_2_O_2_ by-product. An in vitro multi-enzymatic system was successfully established to synthesize the novel insulin secretion accelerant 4-HIL for the treatment of type II diabetes. Moreover, this system is highly efficient at catalyzing reactions involving other hydrophobic aliphatic l-amino acids and could synthesize hydroxy amino acids [28]. Here, Fe(II)/2-KG DOs-mediated C–H oxidation of small molecules was accomplished in vitro using an efficient and sustainable 2-KG supply system that could generate this substrate for other enzymes catalyzing the synthesis of various functional compounds.

## 2. Results and Discussion

### 2.1. Design of a Fe(II)/2-KG DO-Based In Vitro Enzymatic Biosystem for Hydroxy Amino Acid Synthesis

**Figure 1:**
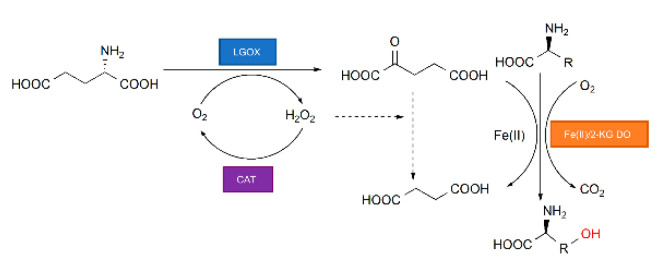
Multi-enzymatic cascade system for hydroxy amino acid synthesis by combining Fe(II)/2-ketoglutarate-dependent dioxygenase (Fe(II)/2-KG DO), l-glutamate oxidase (LGOX), and catalase (CAT).

Fe(II)/2-KG DOs catalyze various reactions including hydroxylation by C–H activation. Binding of the 2-KG co-substrate facilitates substrate binding and forms a ferryl intermediate that is critical for substrate hydroxylation [29]. The quantity of 2-KG directly affects the catalytic efficiency of Fe(II)/2-KG DOs. Thus, we implemented an in vitro bio-cascade reaction system to synthesize hydroxy amino acids by coupling Fe(II)/2-KG DOs-mediated C–H hydroxylation with 2-KG derived from cheap, readily available l-Glu (Figure 1). In this multi-enzymatic cascade system, IDO, the Fe(II)/2-KG DO, catalyzes l-amino acid hydroxylation and synthesizes useful hydroxy amino acids and certain sulfur-containing l-amino acids. This process is accompanied by oxidative 2-KG decarboxylation using Fe^2+^ as a cofactor [28]. LGOX exhibits high specificity for the conversion of l-Glu to 2-KG and simultaneously generates H_2_O_2_. Catalase (CAT) eliminates the H_2_O_2_ [25], thereby preventing its potentially negative effects on the cascade reaction such as the oxidization of 2-KG to succinic acid. Overall, hydroxy amino acid synthesis is accomplished with a simultaneous 2-KG supply by constructing a cell-free reaction system with no additional substrate consumption or membrane-induced mass transfer [30]. Compared with in vivo 2-KG accumulation from the TCA cycle, in vitro cell-free enzymatic system for 2-KG generation has the advantages of high space-time yield and easy operation in product separation. Moreover, this strategy is applicable to other enzymes that catalyze 2-KG-coupled reactions and synthesize other functional compounds.

### 2.2. Enzyme Kinetics

IDO and LGOX in the cascade reaction were purified by His-Trap HP affinity chromatography (Figure S1), and we optimized the reaction conditions to achieve maximum system efficiency (Figure S2). According to the optimized results, we observed the kinetics of IDO and LGOX under 50 mM Tris-HCl, pH 7.0, and 30 °C. In these conditions, the IDO and LGOX activity levels were relatively high. The kinetic parameters were fitted to a Michaelis–Menten model (Table 1). IDO exhibited high oxidative decarboxylation activity on 2-KG (*V*_max_ = 19.79 ± 0.08 U·mg^−1^). It also had relatively high activity in the presence of l-Ile (*V*_max_ = 8.99 ± 0.09 U·mg^−1^). In contrast, l-Ile hydroxylase from *Bacillus cereus* 13658 had an activity of only 0.68 ± 0.06 U·mg^−1^ [31]. LGOX had high specificity and affinity for l-Glu (*K*_m_ = 2.65 ± 0.11 mM and *V*_max_ = 2.93 U·mg^−1^). IDO and LGOX did not markedly differ in terms of *k*_cat_. Therefore, the rate of 2-KG regeneration aligned with that of l-Ile hydroxylation and facilitated 4-HIL synthesis.

### 2.3. Effects of Reaction Components on IDO and LGOX Activity

**Figure 2:**
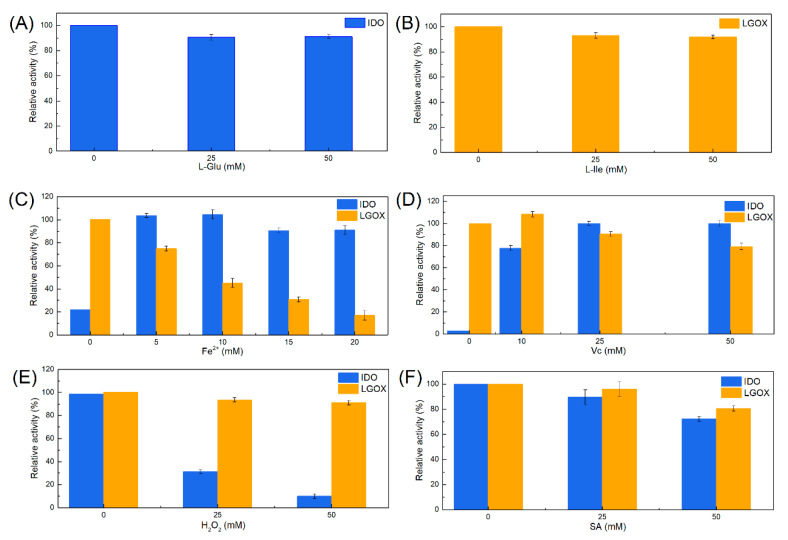
Effects of various reaction components on IDO (blue) and LGOX (orange) activity. IDO and LGOX activity levels were measured using various concentrations of (**A**) l-Glu, (**B**) l-Ile, (**C**) Fe^2+^, (**D**) Vc, (**E**) H_2_O_2_, and (**F**) SA. The specific activities of the enzymes under standard conditions (5.84 ± 0.07 U·mg^−1^ for IDO and 2.67 ± 0.09 U·mg^−1^ for LGOX) were designated as 100%, respectively.

There are generally some factors to be considered for constructing a cascade reaction involving different reaction components [32]. Various intermediates and components may influence the reaction process [33]. We investigated the inhibitory effects of l-Glu, l-Ile, SA, l-ascorbic acid (Vc), Fe^2+^ (FeSO_4_·7H_2_O), and H_2_O_2_ on single enzyme activity by varying their concentrations. Figure 2 shows that H_2_O_2_ strongly inhibited IDO and significantly decreased its activity. Exposure of the enzyme to the oxidizing environment created by H_2_O_2_ oxidized the Fe^2+^ in the IDO active site and decreased enzyme activity. Vc is a reductant that promotes C–H oxidation catalyzed by Fe(II)/2-KG DOs [34]. Vc was required by this system for 4-HIL synthesis. Further, 50 mM SA lowered IDO activity to 74%. However, all other components tested only slightly affected IDO activity. High Fe^2+^ concentrations inactivate LGOX [25]. The relative activity of LGOX was <20% at 20 mM Fe^2+^. IDO is a member of the Fe (II)/2-KG-dependent dioxygenase family. It requires 2-KG and Fe^2+^ as cofactors to catalyze hydroxylation. Here, IDO exhibited little activity towards the substrate even in the absence of Fe^2+^, possibly because of the Fe^2+^ pool in the *E. coli* cells [35]. Therefore, the influence of Fe^2+^ addition on the cascade reaction needs to be further investigated.

### 2.4. Enzymatic Cascade for 4-HIL Synthesis

**Figure 3:**
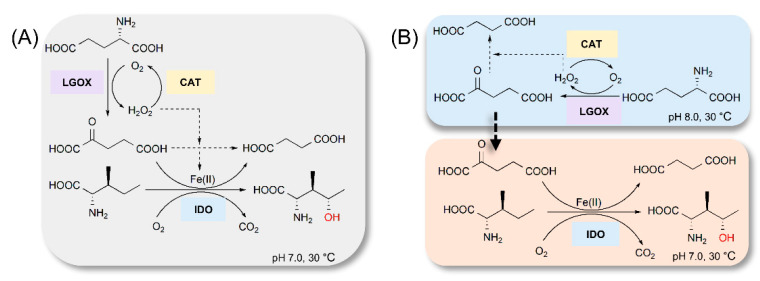
Enzymatic cascade production of 4-HIL. (**A**) Scheme of one-pot production of 4-HIL. (**B**) Scheme of multistep reaction to produce 4-HIL.

Here, we attempted to develop an in vitro system producing hydroxy amino acids and using IDO, LGOX, and CAT as biocatalysts. We tested one-pot 4-HIL production using a combination of the aforementioned enzymes (Figure 3A).

**Figure 4:**
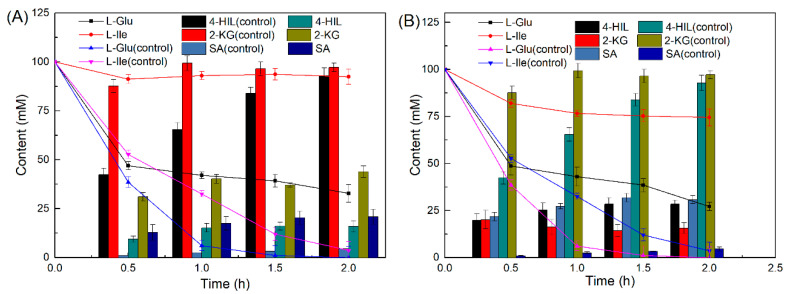
One-pot cascade reaction to produce 4-HIL. (**A**) One-pot cascade reaction in the absence of Fe^2+^; (**B**) one-pot cascade reaction in the presence of Fe^2+^.

We assessed the influence of exogenous Fe^2+^ on 4-HIL production. We mixed 100 mM l-Glu and 100 mM l-Ile in a 25 mL shaker flask and added 1 g·L^−1^ IDO, 0.5 g·L^−1^ LGOX, and 2 mg·L^−1^ CAT to it. Figure 4 shows that 166 mM of 4-HIL was produced in the absence of Fe^2+^ whereas 28 mM of 4-HIL was produced in the presence of Fe^2+^. IDO and LGOX activity obviously decreased in the cascade reaction relative to the single-step reaction (control). The final 4-HIL product did not continue to accumulate after the first hour of the reaction. The reaction mixture presented with low 4-HIL yield (16%) in the absence of Fe^2+^ and low IDO activity (Figure 4A). As shown in Figure 4B, Fe^2+^ obviously inhibited LGOX activity. Minimal l-Glu was consumed and little 4-HIL accumulated after 0.5 h. However, residual 2-KG was detected in the reaction mixture after sufficient CAT was added to eliminate the H_2_O_2_. IDO and LGOX were simultaneously inhibited by H_2_O_2_ and Fe^2^^+^, respectively. Therefore, the generation of 2-KG from l-Glu by LGOX was incompatible with the hydroxylation of l-Ile by IDO in one pot. For this reason, it was preferable to conduct a stepwise cascade reaction to produce hydroxy amino acids efficiently.

**Figure 5:**
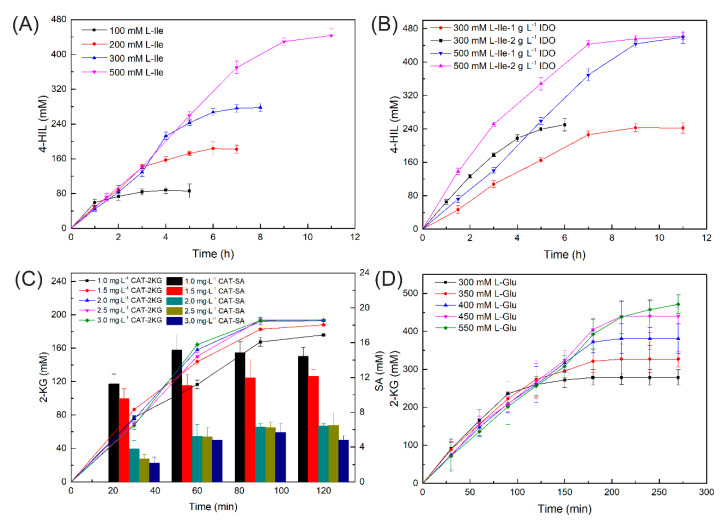
Optimization of biocatalyst and substrate concentrations. (**A**) 4-HIL production at different L-Ile concentrations with 1 g·L^−1^ IDO; (**B**) 4-HIL production at various IDO concentrations; (**C**) 2-KG and SA yield at different CAT concentrations in presence of 0.5 g·L^−1^ LGOX; (**D**) 2-KG yield at various L-Glu concentrations in presence of 0.5 g·L^−1^ LGOX.

We then attempted two-step 4-HIL production (Figure 3B) and further optimized the biocatalyst and substrate concentrations. l-Ile in the concentration range of 100–500 mM was hydroxylated with 1 g·L^−1^ purified IDO. Figure 5A shows that IDO had robust l-Ile hydroxylation activity. We obtained 444 mM of 4-HIL with 500 mM l-Ile as substrate. Thus, the product yield was 89%. Moreover, no substrate inhibition was detected at any substrate concentration. We examined the effects of IDO on l-Ile transformation at various substrate concentrations. The corresponding time courses are shown in Figure 5B. The yield and conversion rate increased with IDO concentration. After 9 h, 2 g·L^−1^ IDO generated 461 mM of 4-HIL from 500 mM l-Ile at a product yield of 92%. After 9 h, 1 g·L^−1^ IDO generated 459 mM of 4-HIL at a product yield of 92%. However, concerning the reaction productivity, 4.9 g·(L·g·h)^−1^ of 4-HIL was obtained with 2 g·L^−1^ IDO, while 7.5 g·(L·g·h)^−1^ of 4-HIL was obtained with 1 g·L^−1^ IDO. Therefore, 1 g·L^−1^ IDO was selected as the catalyst for l-Ile hydroxylation. Hydrogen peroxide is co-produced with 2-KG and may degrade it to SA. The H_2_O_2_ is decomposed to H_2_O and O_2_ by CAT [36]. We explored the effect of varying CAT concentration on 2-KG production using 200 mM l-Glu as the substrate. Figure 5C shows that the SA accumulation was <15 mM in the presence of 2–3 mg·L^−1^ CAT. The 2-KG production slightly increased with increasing CAT concentration. At 2 mg·L^−1^ CAT, 186 mM of 2-KG was generated within 120 min and the yield was 93%. In contrast, only 2.5% SA was formed. No further increase in 2-KG was observed with increasing CAT concentration. We also tested various l-Glu concentrations to establish the optimal reaction conditions (Figure 5D). There was slight substrate inhibition with increasing l-Glu concentration. LGOX generated 491 mM of 2-KG in the presence of 550 mM L-Glu and the yield was 90%.

### 2.5. Fed-Batch 4-HIL Synthesis Reaction

**Figure 6:**
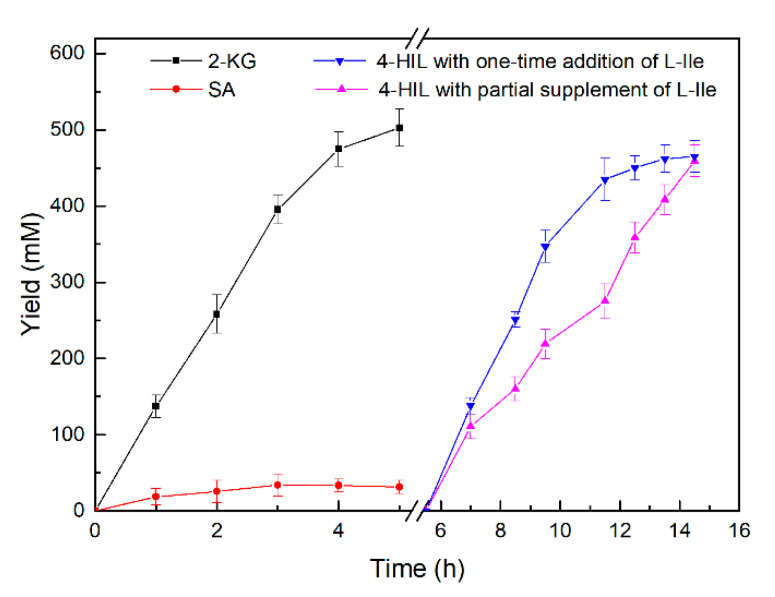
Two-step reaction for 4-HIL production.

L-Ile is the most hydrophobic amino acid. Its solubility is 32.5 g·L^−1^ (247.75 mM) in aqueous solution at 25 °C [37]. To further improve transformation efficiency of cell-free enzymatic system for 4-HIL synthesis, we explored methods of feeding l-Ile in a 500 mL bioreactor (Figure 6). l-Glu at 550 mM was fully converted to 2-KG in ~5 h and only <35 mM SA accumulated. In the first oxidation reaction, 491 mM of 2-KG was obtained at a reaction rate of 14.5 g·L^−1^·h^−1^. Optimal concentrations of the l-Ile hydroxylation components were added to the system and 500 mM l-Ile was added either in one dose or in batches at an initial concentration of 200 mM. IDO maintained robust l-Ile hydroxylation activity when the substrate was added in one dose. We obtained 434 mM of 4-HIL after 6 h of l-Ile hydroxylation. The l-Ile hydroxylation reaction rate significantly decreased when the initial substrate concentration was 200 mM as the l-Ile was rapidly consumed. Nevertheless, the reaction rate increased after substrate supplementation. In the second hydroxylation reaction, when l-Ile was incrementally supplemented, IDO yielded 440 mM of 4-HIL after 9 h. In contrast, IDO produced 465 mM of 4-HIL after the l-Ile was added in one dose. The total reaction time for the entire cascade reaction was 14.5 h. The first step produced 491 mM of 2-KG from l-Glu within 5 h and the yield was 89%. The second step generated 464.96 mM of 4-HIL after 9 h when l-Ile was added in one dose. The yield was 93%. Hence, it is feasible to scale up the foregoing 4-HIL production system. In contrast, an in vivo 2-KG supply for 4-HIL synthesis has been successfully implemented by redirecting the TCA cycle [21,38]. However, metabolic engineering strategies of shifting the carbon flux from l-Ile to 2-KG by boosting the TCA cycle and further increasing the pool of oxaloacetate were adopted to obtain 34.21 g/L (232.45 mM) of 4-HIL at 64 h, inevitably hindering normal cell growth [21]. The cell-free enzymatic system generating 2-KG from l-Glu was favorable for 4-HIL production with high substrate concentration and space–time yield. Furthermore, the methodology optimized here could lay the foundation for the development of other industrial bioprocess technologies.

### 2.6. In Vitro Enzymatic Biosystem for Hydroxy Amino Acids Synthesis

**Figure 7:**
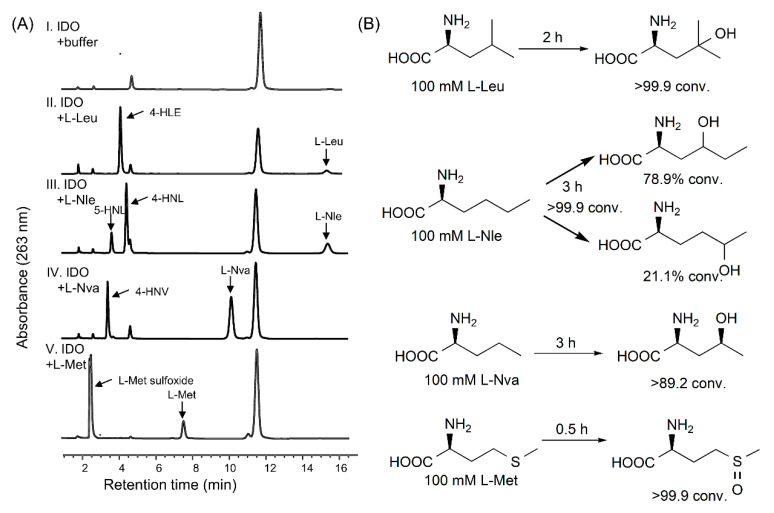
In vitro enzymatic synthesis of hydroxy amino acids. (**A**) Hydroxy amino acids were generated from a two-step system using various l-amino acids as substrates. Products were analyzed by LC-MS. (**B**) Biotransformation data for the productions of different hydroxy amino acids.

Hydroxy amino acids are active ingredients or building blocks in pharmaceutical synthesis. Thus, effective, sustainable hydroxy amino acid production is urgently required. IDO has enzymatic activity towards certain hydrophobic aliphatic l-amino acids and sulfur-containing amino acids [28]. It hydroxylates or sulfonates C–H bonds and generates hydroxy amino acids and l-amino acids sulfoxides, respectively. Here, we used 100 mM of l-amino acids substrates in the presence of IDO. All enzymatic reactions achieved high substrate conversion rates. Figure 7 shows that LGOX generated 102.19 mM of 2-KG from l-Glu within 1 h. IDO produced 16.47 g·L^−1^ of l-methionine sulfoxide (>99.9% conversion) within the first 0.5 h in the presence of l-methionine (l-Met). IDO generated 14.61 g·L^−1^ of 4-hydroxy-l-leucine (>99.9% conversion) within 2 h in the presence of l-leucine (l-Leu). IDO converted 89.2% of l-norvaline (l-Nva) after 3 h. When l-norleucine (l-Nle) was the substrate, the putative diastereomers 4-hydroxy-l-norleucine and 5-hydroxy-l-norleucine were detected. The reaction products were characterized by mass spectrometry (Figure S3). The aforementioned reactions demonstrated that the application of a two-step cascade system was highly efficient for the production of hydroxy amino acids and l-amino acid sulfoxides via direct C–H functionalization.

## 3. Materials and Methods

### 3.1. Reagents

The (2*s*,3*r*,4*s*)-4-hydroxyisoleucine standard was purchased from Cambridge Sigma-Aldrich (Munich, Germany). All other analytical-grade chemicals were obtained from Sinopharm Chemical Reagent Co. (Shanghai, China). All enzymes for genetic manipulations were obtained from TaKaRa Biotechnology Co. (Dalian, China). Protein purification columns were acquired from GE Healthcare (Munich, Germany).

### 3.2. Preparation of Recombinant Enzymes

Commercial catalase (CAT) was obtained from Aladdin Bio-Chem Technology Co. Ltd. (Shanghai, China). The 6×His-tagged IDO-expressing strain (*E. coli* BL21(DE3)-IDO) [28] and 6×His-tagged LGOX-expressing strain (*E. coli* BL21(DE3)-LGOX) [25,26] were constructed as previously described. IDO and LGOX precultures were incubated overnight in 5 mL of Luria–Bertani (LB) medium (Sangon Biotech, Shanghai, China) with 50 μg·mL^−1^ kanamycin (Sinopharm Chemical Reagent, Shanghai, China) at 37 °C on a rotary shaker (200 rpm). IDO and LGOX were induced with 0.5 mM isopropyl-β-d-1-thiogalactopyranoside (IPTG) in 200 mL of LB medium containing 50 μg·mL^−1^ kanamycin. The cultures were incubated for 3 h at 37 °C and then for 16 h at 17 °C.

The cells were harvested by centrifugation (10,800× *g*, 4 °C, 5 min) and resuspended in 20 mM Tris-HCl buffer (pH 7.5). The bacterial pellet was disrupted and centrifuged (10,800× *g*, 4 °C, 40 min). The recombinant IDO and LGOX supernatants were purified by Ni-affinity chromatography as previously reported [39]. The supernatant was purified in a His-Trap HP affinity column (GE Healthcare, Little Chalfont, UK) [40]. The elution buffer in the affinity columns created a high salt content in the purified fractions. The excess salt was removed with disposable PD-10 desalting columns (GE Healthcare, Little Chalfont, UK) [41]. The proteins were analyzed by sodium dodecyl sulfate-polyacrylamide gel electrophoresis (SDS-PAGE) [42] and the yields were determined by NanoDrop 8000 Microvolume UV-Vis spectrophotometry (Thermo Fisher Scientific, Waltham, MA, USA).

### 3.3. Enzymatic Activity Assays

For IDO, a mixture of 10 mM l-Ile, 1.5 mM FeSO_4_, 10 mM l-ascorbic acid, 10 mM 2-KG, and 50 mM Tris-HCl buffer (pH 7.0) was incubated in a Thermomixer Comfort incubator (Eppendorf; Hamburg, Germany) at 30 °C with 800 rpm agitation. The reaction was initiated with 0.1 g·L^−1^ purified IDO in a final volume of 300 µL [43]. Samples were collected at 0, 5, and 10 min after enzyme addition and analyzed by HPLC [44]. One unit of enzymatic activity was defined as the amount of enzyme required to catalyze the synthesis of 1 µmol of 4-HIL min^−1^ under standard conditions.

For LGOX, a mixture of 10 mM l-Glu, 1 mg·L^−1^ CAT, and 50 mM Tris-HCl buffer (pH 7.0) was incubated in a Thermomixer Comfort incubator (Eppendorf; Hamburg, Germany) at 30 °C, with 800 rpm agitation. The reaction was initiated with 0.1 g·L^−1^ purified LGOX in a final volume of 300 µL. Samples were collected at 1, 3, and 5 min after enzyme addition and analyzed by HPLC. One unit of enzymatic activity was defined as the amount of enzyme required to catalyze the synthesis of 1 µmol of 2-KG· min^−1^ under standard conditions.

### 3.4. Determination of K_m_ and V_max_

For IDO, the optimal concentration of the cascade reaction was used and the l-Ile concentration range was 0.5–20 mM. The concentration of 2-KG was in the range of 1–40 mM. The kinetic parameter assays were conducted in triplicate. For LGOX, the optimal concentration of the cascade reaction was used and the l-Glu concentration range was 0.5–20 mM. IDO and LGOX activity were measured as previously described. The kinetic parameters were fitted to a Michaelis–Menten model [45].

### 3.5. Effects of Reaction Components on Enzyme Activities

The inhibitory effects of l-Ile, l-Glu, Fe^2+^, Vc, H_2_O_2_, and SA were tested by comparing the initial reaction rates of IDO and LGOX in the presence of various concentration of the aforementioned compounds. IDO and LGOX activity were measured as previously described.

### 3.6. One-Pot Synthesis of 4-HIL

The reaction mixture consisted of 100 mM l-Ile, 50 mM Vc, 100 mM l-Glu, 1 g·L^−1^ IDO, 0.5 g·L^−1^ LGOX, 2 mg·L^−1^ CAT, and 50 mM Tris-HCl buffer (pH 7.0). Then 5 mM FeSO_4_·7H_2_O was added to the mixture to compare the effects of Fe^2+^ on the cascade reaction. The system comprised 5 mL of the reaction mixture heated to 30 °C in a 25-mL shaker flask rotating at 200 rpm.

### 3.7. Two-Step Process for Hydroxy Amino Acid Synthesis

For the 2-KG regeneration step, the reaction mixture consisted of 0.5 g·L^−1^ LGOX, various concentrations of l-Glu (300–550 mM) and CAT (1-3 mg·L^−1^), and 50 mM Tris-HCl buffer (pH 8.0) in a final volume of 5 mL. The reaction was conducted at 30 °C and 200 rpm. For the l-amino acid hydroxylation step, l-amino acids hydroxylation by IDO was examined after 2-KG production by LGOX. The reaction mixture comprised 5 mM FeSO_4_, 50 mM Vc, 1 g·L^−1^ IDO, and 50 mM Tris-HCl buffer (pH 7.0) in a final volume of 5 mL. Using various substrate concentrations (100–500 mM), the 2-KG obtained from the first step was added in equimolar concentrations with the l-amino acids. The mixture was incubated in a shaker flask at 30 °C and 200 rpm.

### 3.8. Fed-Batch Reaction for 4-HIL Synthesis

For 2-KG generation, the reaction mixture was added to a 500-mL reactor consisting of 0.5 g·L^−1^ LGOX, 550 mM l-Glu, 2 mg·L^−1^ CAT, and 50 mM Tris-HCl buffer (pH 8.0) in a final volume of 100 mL. The reaction was conducted at 30 °C and 400 rpm. After 5 h, the components containing 10 mM FeSO_4_, 50 mM Vc, and 1 g·L^−1^ IDO were mixed into the reactor. The pH was fixed at 7.0. The one-dose and incremental L-Ile addition methods were further compared as l-Ile has low solubility in aqueous solvents. l-Ile 500 mM was added to the reactor in one dose to synthesize 4-HIL. The fed-batch reaction was conducted using 200 mM l-Ile as the initial concentration. Then 150 mM l-Ile was supplemented every 2 h until a final concentration of 500 mM l-Ile was reached. The reactions were conducted at 30 °C and 400 rpm. The supernatants were sampled every hour and analyzed.

### 3.9. Analysis of Organic Acids

The organic acids in the samples were analyzed in a Waters 2695 HPLC system (Waters Co., Milford, MA, USA) fitted with an Atlantis T3 column (4.6 mm × 250 mm). Compounds were detected at 210 nm using an injection volume of 10 µL and a column temperature of 40 °C. The mobile phase was 20 mM phosphate buffer (pH 2.8) and the flow rate was 0.8 mL·min^−1^.

### 3.10. Analysis of Amino Acids

The samples were analyzed in a Waters 2695 HPLC system (Waters Co., Milford, MA, USA) fitted with a Diomansil C18 column (4.6 mm × 250 mm). The chromatographic conditions were as follows: mobile phase A (NaAc-HAc buffer (50 mM, pH 4.2):acetonitrile = 50:50); mobile phase B (acetonitrile); gradient elution program; flow rate, 1 mL min^−1^; column temperature, 25 °C; injection volume, 10 µL. Post-column derivatization was conducted with Fmoc-Cl [44]. The Fmoc-Cl derivatives of the amino acids were detected at 263 nm.

LC-MS analysis was conducted in a Waters ACQUITY UPLC-MS system fitted with a Waters ACQUITY UPLC HSS C18 reverse-phase column (inner diameter, 1.8 µm) (Waters Co., Milford, MA, USA). The inlet, MS transfer line, and ion source temperatures were set to 280, 280, and 230 °C, respectively.

### 3.11. Nuclear Magnetic Resonance (NMR) Analysis

All products were isolated by cation exchange chromatography using a strong cation resin (C100E (H^+^ form), Purolite, King of Prussia, PA, USA) [46]. l-Ile hydroxylation generated 40 mM of 4-HIL and 42% yield. The 4-HIL spectrum was recorded by ^1^H-NMR spectroscopy (Figure S4) as previously described [9].

(2*s*,3*r*,4*s*)-4-hydroxyisoleucine: δ 3.84 (d, J = 4.4 Hz, 1H), 3.79 (dd, J = 13.6, 6.8 Hz, 1H), 1.92–1.82 (m, 1H), 1.19 (d, J = 6.3 Hz, 3H), 0.91 (d, J = 7.1 Hz, 3H).

## 4. Conclusions

Here, we developed an in vitro enzymatic system to synthesize hydroxy amino acids via Fe(II)/2-KG DOs-mediated C–H bond oxidation. Amino acid hydroxylation by Fe(II)/2-KG DO was coupled with generation of the prerequisite 2-KG from inexpensive and abundant l-Glu. After systematically assessing the catalytic parameters of the catalysts, a sequential cascade reaction was preferable for the efficient synthesis of hydroxy amino acids. Optimizing the catalyst and substrate concentrations in the two-step process resulted in 465 mM of 4-HIL and 93% yield. Otherwise, IDO was highly active towards other hydrophobic aliphatic l-amino acids and could, therefore, produce other hydroxy amino acids from them. Hence, this system is promising for in vitro biosynthesis of hydroxy amino acids, and more efforts should be necessary to enhance the resistance of IDO and LGOX towards the inhibition of reaction generated components, so as to facilitate the one-pot reaction system. It is also a viable alternative to other Fe(II)/2-KG DO-mediated C–H bond functionalization strategies for the synthesis of functional compounds.

## Figures and Tables

**Table 1:** Kinetic parameters of IDO and LGOX.

Enzyme	Substrate	*K*_m_ (mM)	*V*_max_ (U·mg^−1^)	*k*_cat_ (s^−1^)	*k*_cat_/*K*_m_ (s^−1^·m·M^−1^)
IDO	l-Ile	6.34 ± 0.12	8.99 ± 0.09	4.18 ± 0.08	0.66 ± 0.04
	2-KG	15.12 ± 0.08	19.79 ± 0.08	9.18 ± 0.07	0.61 ± 0.07
LGOX	l-Glu	2.65 ± 0.11	2.93 ± 0.06	3.38 ± 0.05	1.28 ± 0.07

Kinetic parameters were measured in 50 mM Tris-HCl (pH 7.0) at 30 °C.

## Supplementary Materials

Supplementary materials can be found at https://www.mdpi.com/1422-0067/21/15/5347/s1.
