# Supplementary material for: 2-Ketoglutarate-Generated In Vitro Enzymatic Biosystem Facilitates Fe(II)/2-Ketoglutarate-Dependent Dioxygenase-Mediated C–H Bond Oxidation for (2s,3r,4s)-4-Hydroxyisoleucine Synthesis
Source: Int J Mol Sci. 2020 Jul 28;21(15):5347. doi: 10.3390/ijms21155347 (PMC7432852; doi:10.3390/ijms21155347)
Supplement: Supplementary file 1 [file ijms-21-05347-s001.pdf]

International Journal of
Molecular Sciences

MDPI

Article

2-Ketoglutarate-generated in vitro enzymatic biosystem facilitates Fe(II)/2-ketoglutarate-dependent dioxygenase-mediated C–H bond oxidation for (2*s*,3*r*,4*s*)-4-Hydroxyisoleucine synthesis

Xiaoran Jing[a], Huan Liu[a], Yao Nie[a]*, Yan Xu[a,b]*

a. Key Laboratory of Industrial Biotechnology of Ministry of Education and School of Biotechnology, Jiangnan University, 1800 Lihu Road, Wuxi 214122, China

b. State Key Laboratory of Food Science and Technology, Jiangnan University, 1800 Lihu Road, Wuxi 214122, China

*E-mail: ynie@jiangnan.edu.cn (Y. Nie); yxu@jiangnan.edu.cn (Y. Xu)

1. SDS-PAGE analysis of purified enzymes

The recombinant enzymes were purified by His-Trap HP affinity chromatography using a His-Trap HP affinity column, then the high salt content of the purified fractions was removed using a disposable PD-10 desalting column.

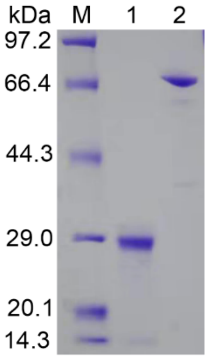

Figure S1. Sodium dodecyl sulfate–polyacrylamide gel electrophoresis (SDS-PAGE) analysis of purified enzymes: M: Molecular weight standards (Bio-Rad); lane 1: Purified L-isoleucine dioxygenase (IDO); lane 2: Purified L-glutamate oxidase (LGOX).

2. *Effects of pH and temperature on enzyme activity*

To determine the effects of pH on enzyme activity, we separately measured the IDO and LGOX activity in the standard reaction mixture at a 4.0–9.0 pH range. The effects of temperature on IDO and LGOX activity were evaluated by incubating a standard reaction mixture for 10 min at temperatures ranging from 10–45 °C. Figure S2A shows that IDO and LGOX activity were at a maximum at 30 °C. Therefore, the enzyme cascade temperature was set to 30 °C. Figure S2B shows IDO and LGOX activity with respect to pH and buffer. IDO had maximum activity in Tris-HCl buffer (pH 7.0), whereas LGOX had 93% activity in Tris-HCl buffer (pH 8.0). IDO had long-term stability at 25–35 °C (Figure 2C) while the relative LGOX activity was >90% after 3 h incubation at 25 °C and 30 °C. However, LGOX was unstable at 35 °C (Fig. 2D). Thus, Tris-HCl buffer (pH 7.0) and 30 °C were applied in one-pot 4-HIL production.

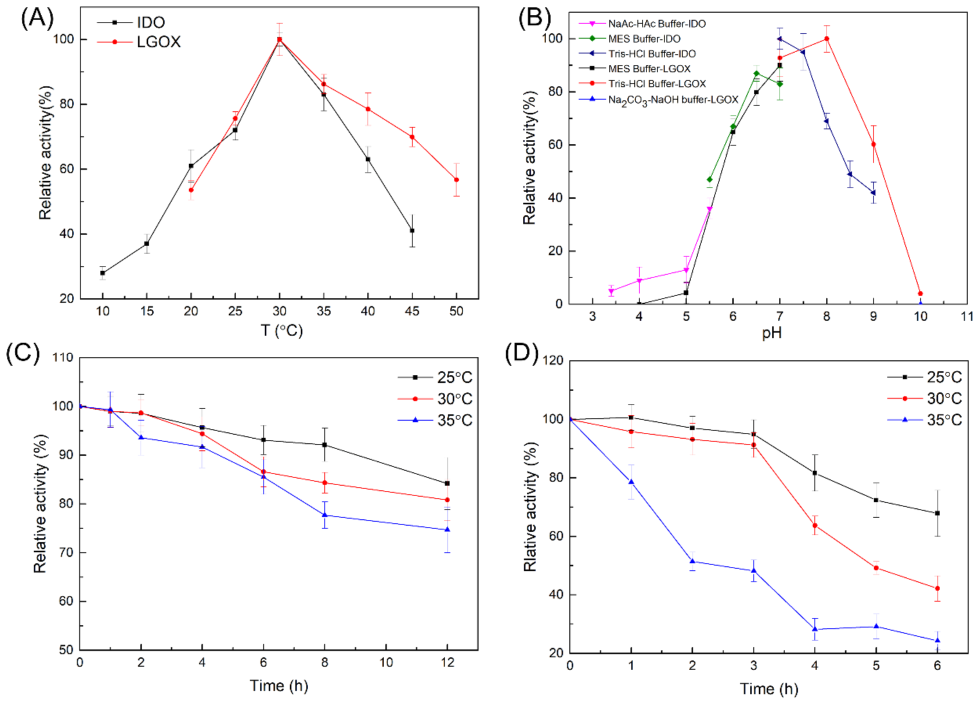

Figure S2. Effects of reaction conditions on IDO and LGOX activity. (**A**) Activity profiles of IDO and LGOX as functions of temperature; (**B**) activity profiles of IDO and LGOX as functions of pH and buffer; (**C**) IDO thermostability; (**D**) LGOX thermostability.

3. LC-MS analysis

LC-MS analysis of five products were recorded on the MALDI SYNAPT MS SYSTEM-WATERS instrument.

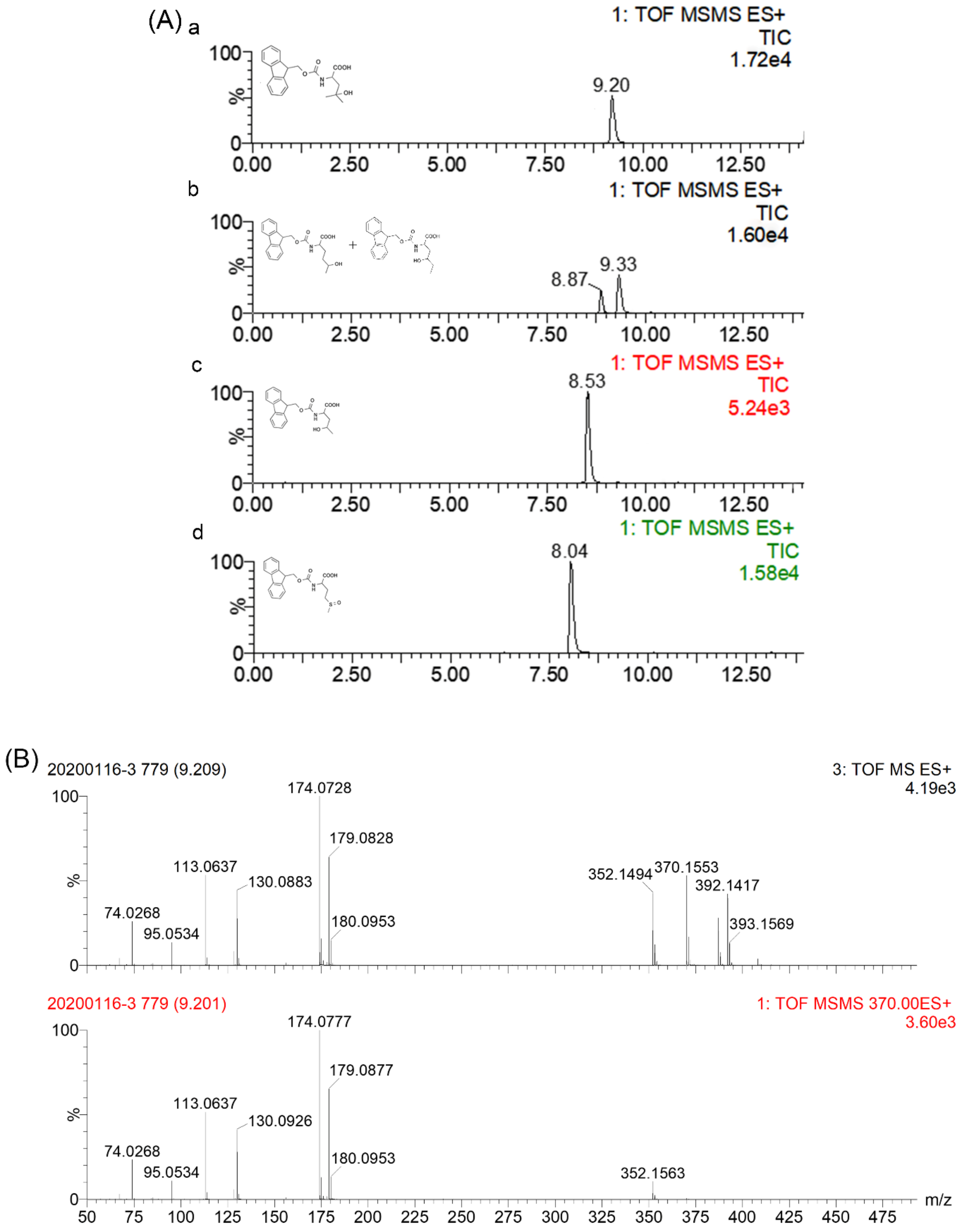

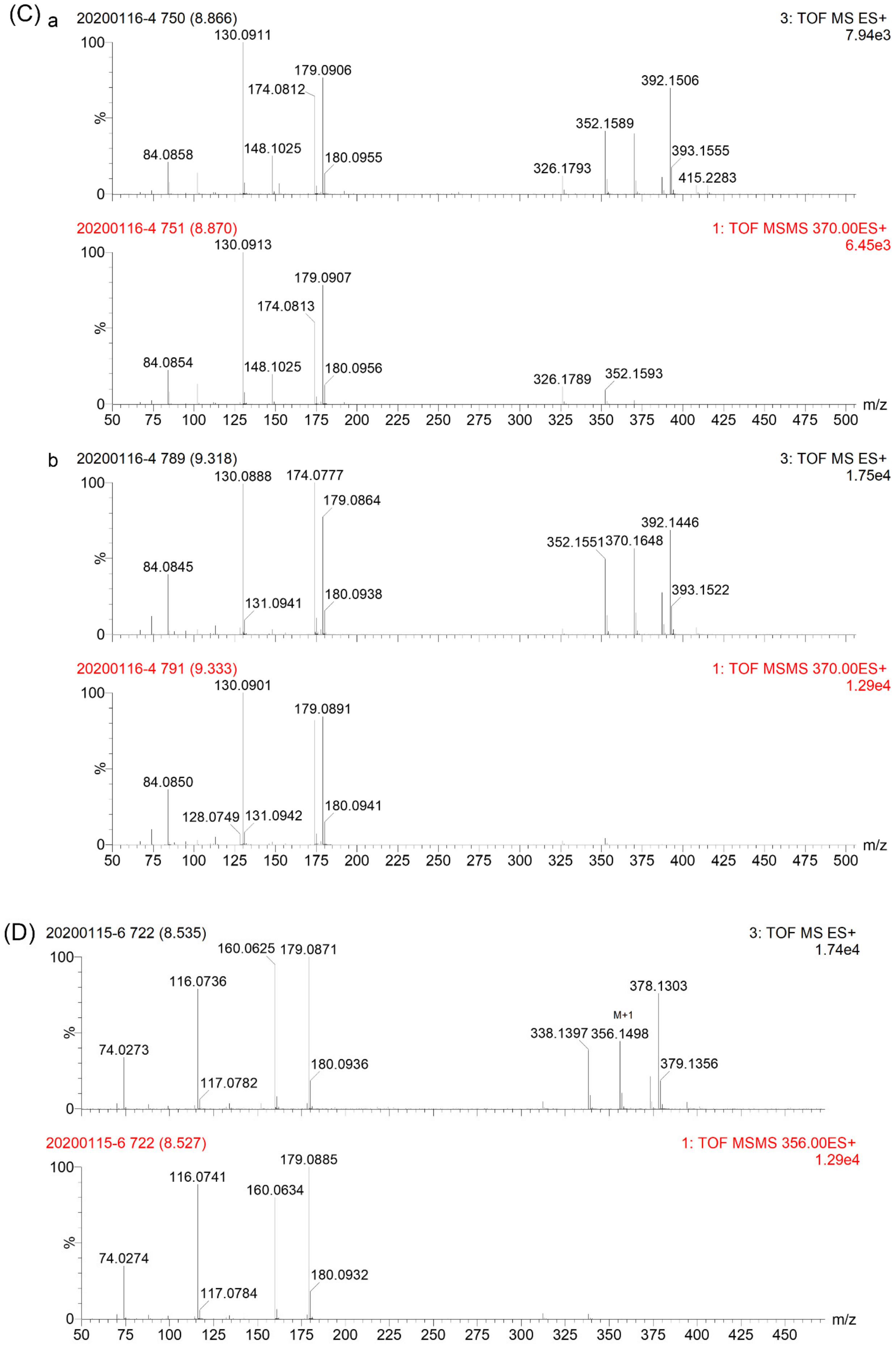
(C) a
20200116-4 750 (8.866)
3: TOF MS ES+
7.94e3
130.0911
179.0906
174.0812
392.1506
352.1589
84.0858
148.1025
180.0955
326.1793
393.1555
415.2283
20200116-4 751 (8.870)
1: TOF MSMS 370.00ES+
6.45e3
130.0913
179.0907
174.0813
84.0854
148.1025
180.0956
326.1789
352.1593
m/z
b
20200116-4 789 (9.318)
3: TOF MS ES+
1.75e4
130.0888
174.0777
179.0864
392.1446
352.1551
370.1648
84.0845
180.0938
131.0941
393.1522
20200116-4 791 (9.333)
1: TOF MSMS 370.00ES+
1.29e4
130.0901
179.0891
84.0850
128.0749
131.0942
180.0941
m/z
(D)
20200115-6 722 (8.535)
3: TOF MS ES+
1.74e4
160.0625
179.0871
116.0736
378.1303
M+1
338.1397
356.1498
74.0273
180.0936
117.0782
379.1356
20200115-6 722 (8.527)
1: TOF MSMS 356.00ES+
1.29e4
179.0885
116.0741
160.0634
74.0274
180.0932
117.0784
m/z
%
100
0
50 75 100 125 150 175 200 225 250 275 300 325 350 375 400 425 450 475 500

(E)

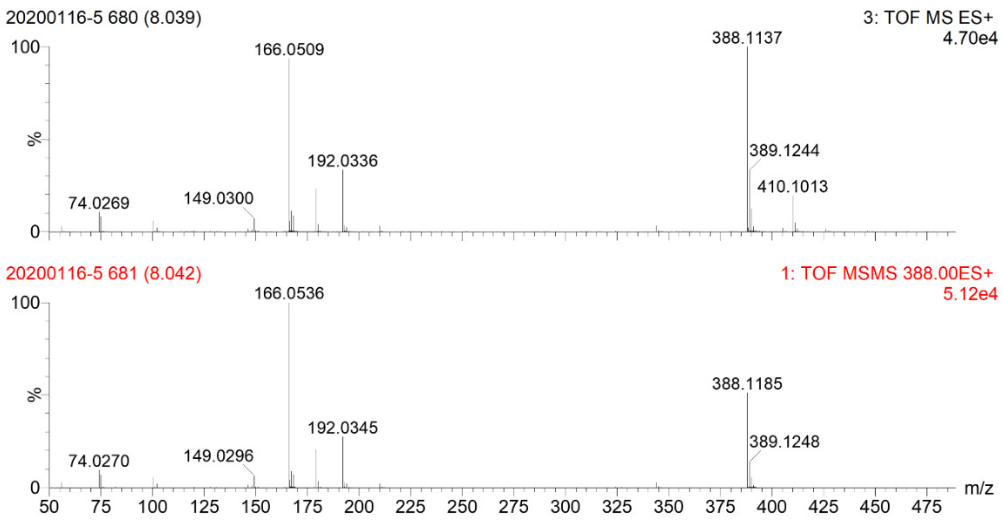

Figure S3. Products analysis by LC-MS. (**A**) Hydroxyl amino acids were generated from the two-step system with different L-amino acids as substrates, where the derivatives of five products by Fmoc-Cl were analyzed by LC-MS; (**B**) mass spectrometry analysis of the derivative of 4-hydroxy-L-leucine by Fmoc-Cl; (**C**) mass spectrometry analyses of the derivatives of 5-hydroxy-L-norleucine (a) and 4-hydroxy-L-norleucine (b) by Fmoc-Cl; (**D**) mass spectrometry analysis of the derivative of 4-hydroxy-L-norvaline by Fmoc-Cl; (**E**) mass spectrometry analysis of the derivative of L-methionine sulfoxide by Fmoc-Cl.

4. Nuclear magnetic resonance (NMR) analysis of (2S,3R,4S)-4-hydroxyisoleucine (4-HIL)

^{1}H NMR of 4-HIL was recorded on Avance 400 (Bruker, Billerica, MA, USA) at 400 MHz (^{1}H NMR). Chemical shifts were reported in ppm down field from internal Me4Si. Multiplicity was indicated as follows: s (singlet), d (doublet), t (triplet), q (quartet), m (multiplet), dd (doublet of doublet), br (broad). Coupling constants were reported in Hertz (Hz).

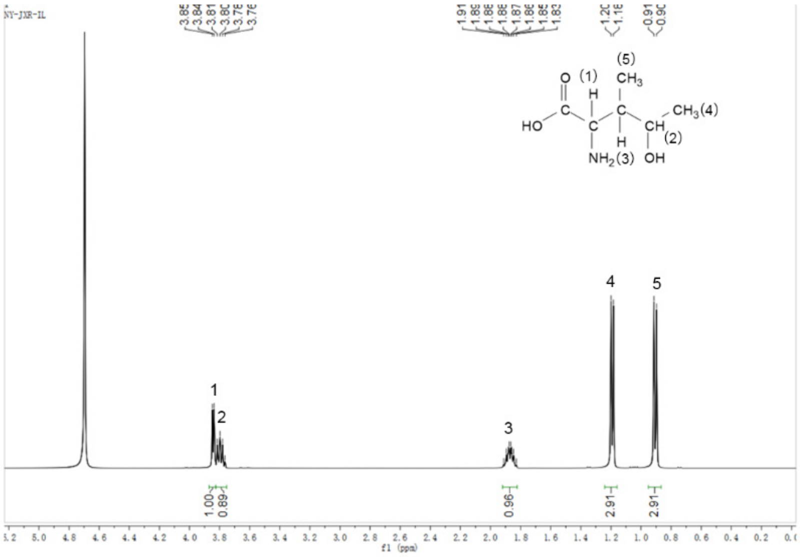

^{1}H NMR (400 MHz, D_2O) δ 3.84 (d, J = 4.4 Hz, 1H), 3.79 (dd, J = 13.6, 6.8 Hz, 1H), 1.92 – 1.82 (m, 1H), 1.19 (d, J = 6.3 Hz, 3H), 0.91 (d, J = 7.1 Hz, 3H).

Figure S4. Analysis of 4-HIL generated from L-isoleucine hydroxylation by ^{1}H-NMR spectrum.

© 2020 by the authors. Submitted for possible open access publication under the terms and conditions of the Creative Commons Attribution (CC BY) license (http://creativecommons.org/licenses/by/4.0/).
